# Synthesis of α-Fe_2_O_3_/Bi_2_WO_6_ layered heterojunctions by *in situ* growth strategy with enhanced visible-light photocatalytic activity

**DOI:** 10.1038/s41598-019-43917-w

**Published:** 2019-05-17

**Authors:** Taiping Xie, Yue Liu, Haiqiang Wang, Zhongbiao Wu

**Affiliations:** 1 Key Laboratory of Environment Remediation and Ecological Health, Ministry of Education, Department of Environmental Engineering, Zhejiang University, 866 Yuhangtang Road, Hangzhou, 310058 P.R. China; 2 Zhejiang Provincial Engineering Research Center of Industrial Boiler & Furnace Flue Gas Pollution Control, 866 Yuhangtang Road, Hangzhou, 310058 P.R. China

**Keywords:** Pollution remediation, Photocatalysis

## Abstract

Layered heterojunction structure with larger interface region for electron migration has attracted much attention in recent years. In this work, layered α-Fe_2_O_3_/Bi_2_WO_6_ heterojunctions with strong interlayer interaction were successfully synthesized through a facile *in situ* growth method. The strong interaction between α-Fe_2_O_3_ and Bi_2_WO_6_ had resulted in excellent photoelectrochemical performance. It was found that such structure promoted the interfacial photogenerated charges separation according to EIS and Tafel analysis, except for the expansion of visible-light absorption range. PL and TRPL characterizations further demonstrated that the recombination ratio of photoexcited electron-hole pairs was greatly reduced. The toluene photocatalytic degradation tests had showed that α-Fe_2_O_3_/Bi_2_WO_6_ composites exhibited much well activity under visible-light irradiation. Especially, 4%-Fe_2_O_3_/Bi_2_WO_6_ sample displayed the highest photocatalytic activity, which was around 3 and 4 times higher than that of pure Bi_2_WO_6_ and α-Fe_2_O_3_. Based on ESR results and free radical trapping experiments, hydroxyl radicals (**·**OH) and holes (h^+^) were regarded as the main active species. The establishment of Fe_2_O_3_/Bi_2_WO_6_ with layered heterojunctions could provide new insights into the construction of novel photocatalysts.

## Introduction

In recent years, indoor air pollution caused by volatile organic pollutants (VOCs) has attracted lots of public attentions. Photocatalytic oxidation was considered as an environmental-friendly technology for indoor VOCs purification^1–6^. Therefore, many kinds of photocatalysts have been extensively investigated, such as TiO_2_^7–9^, ZnO^10,11^, SnO_2_^12^, SrTiO_3_^13^ and so on. However, these semiconductors still have some common shortcomings, such as narrow light absorption range and high recombination ratio of photogenerated charges^14^. Thus, developing visible-light driven and highly active photocatalysts is one of the most urgent topics.

N-type Bi_2_WO_6_ has widely regarded as a promising photocatalyst for its outstanding photooxidation ability, nontoxicity, well thermal and chemical stability^15,16^. Nevertheless, pure Bi_2_WO_6_ can’t efficaciously utilize visible light due to the fact that it can only be driven by light shorter than 450 nm^17,18^. Moreover, low separation ratio of photogenerated electrons and holes also impedes its application. Semiconductor heterojunction has been demonstrated to be an effective way to solve this issue above, such as the coupling of MoS_2_/Bi_2_WO_6_^19^, g-C_3_N_4_/Bi_2_WO_6_^20^, CeO_2_/Bi_2_WO_6_^21^, BiOBr/Bi_2_WO_6_^22^ and so on.

Layered heterojunction photocatalyst possesses larger interfacial area compared to line contact and point contact heterojunction photocatalysts, which benefits the transfer of photogenerated electron-hole pairs^23^. By taking this advantage into consideration, many photocatalysts with layered heterostructure have been fabricated, such as SnS_2_/g-C_3_N_4_^23^, g-C_3_N_4_/Bi_2_WO_6_^24^, g-C_3_N_4_/Bi_20_TiO_32_^25^, α-Fe_2_O_3_/graphene^26^ and so on. Motivated by the above work, Bi_2_WO_6_ coupled with α-Fe_2_O_3_ (a low-price and narrow band gap semiconductor) nanosheets as layered heterojunction photocatalyst may exhibit significantly enhanced photoinduced interfacial charge transfer rate, which could effectively promote the photocatalytic activity.

In this work, layered α-Fe_2_O_3_/Bi_2_WO_6_ heterojunctions were fabricated via a facile *in situ* growth hydrothermal method. And toluene was chosen as a typical kind of indoor VOCs in the experiment. Then, the photodegradation efficiency of gaseous toluene under visible light irradiation was tested. After that, the inherent structure-performance relationship was then disclosed based on the physiochemical and photo-electrochemical characterizations. The main purpose of our work was to shed new light on the synthesis of layered heterojunctions and reveal the role of such structure in photocatalytic process.

## Experimental

### Chemicals

Bi(NO_3_)_3_**·**5H_2_O, Na_2_WO_4_**·**2H_2_O, FeCl_3_**·**6H_2_O, Na_2_SO_4_, NH_3_**·**H_2_O, Na_2_C_2_O_4_, salicylic acid, benzoquinone, sodium acetate and ethanol were purchased from Sigma-Aldrich. All chemical reagents were of analytical grade and without any further purification.

### Synthesis of α-Fe_2_O_3_/Bi_2_WO_6_ composites

Bi_2_WO_6_ was synthesized via the same method as reported in our previous work^15^. Layered α-Fe_2_O_3_/Bi_2_WO_6_ heterojunctions were synthesized by *in situ* growth method. Typically, 2 g Bi_2_WO_6_ was ultrasonic dispersed in 120 mL ethanol for 30 minutes. Then, appropriate amount of FeCl_3_**·**6H_2_O and sodium acetate were added into the above solution. After vigorous stirring for about 2 h, the mixture was transformed to a 200 mL Teflon-lined autoclave and then heated in an oven at 180 °C for 24 h. The obtained participates were collected by vacuum filtration and washed with deionized water and ethanol several times. Finally, these samples were dried in air at 70 °C before being used. Composites of 2, 4 and 6% α-Fe_2_O_3_/Bi_2_WO_6_ samples were prepared, respectively. Pure hexagonal nanoplates of α-Fe_2_O_3_ were synthesized without the addition of Bi_2_WO_6_ via the method reported in the reference^26^.

### Characterization of samples

The crystal phase and composition of these as-prepared catalysts were obtained using an X-ray diffraction (XRD, model D/max RA, Rigaku Co., Japan with Cu Kα radiation). Raman measurement was performed using a LABRAM-HR Ramas-cope fitted with a spectra physics argon ion laser. Laser radiation (λ = 514 nm) was used as excitation source at 20 mW. The surface properties were performed using X-ray photoelectron spectroscopy (XPS) measurement (Thermo, ESCALAB 250). The standard binding energy of 284.8 eV from C1s value was chosen as a reference. The morphology and microstructure information of the catalysts were obtained by Scanning electron microscopy (SEM, FEI-quanta 200F, USA) and transmission electron microscopy (TEM, H-600, Hitachi, Ltd., Japan). The specific surface area of catalysts was ascertained by using a nitrogen adsorption apparatus (Beijing JWGB Sci. & Tech. Co., Ltd). The light adsorption ability of these samples was obtained by using a Scan UV-visible spectrophotometer (UV-visible DRS: TU-1901, China) equipped with an integrating sphere assembly. The spectra were recorded at room temperature in air, ranging from 230 to 850 nm. Photoluminescence (PL) spectra and time-resolved photoluminescence (TRPL) spectra were recorded using a fluorospectrophotometer (PL: RAMANLOG 6, USA) with a 390 nm Ar^+^ laser as excitation source. All these photoelectrochemical properties of the samples were measured on an electrochemical system (CHI 660B, Shanghai, China) using a three-electrode photo electrochemical cell. Platinum wire and saturated Ag/AgCl electrode were used as the counter electrode and the reference electrode, respectively. The working electrode was composed of indium tin oxide (ITO, 20 × 30 × 1.1 mm, 15 Ω) with an area of about 1 cm^2^, glass coated with the prepared samples. The details of preparing working electrode was reported in our previous work^15^. The electrolyte was 0.2 M Na_2_SO_4_ solution. Electron spin resonance (ESR) signals of radical species trapped by 5, 5-dimethyl-1-pyrroline N-oxide (DMPO) were detected on a JES FA200 spectrometer.

### Photocatalytic activity tests

Photocatalytic activity of as-prepared samples was evaluated via photodegradation of toluene. Experiment was performed in a 1.5 L batch reactor sealed with quartz plate. Circulating cooling water in the jacket around the reactor was used to control the reaction temperature. For each test, 0.1 g photocatalyst was uniformly dispersed onto a glass dish with a diameter about 10 cm. After that, the catalyst-coated dish was played on the bottom of the reactor. An appropriate amount of toluene was injected into the reactor with a micro-springe. Before each test, the system was maintained in the darkness to achieve adsorption-desorption equilibrium. The initial concentration of toluene was controlled at about 25 ppm. A 300 W Xe lamp (Celhx300UV, Ceaulight, China) equipped with two optical glass filters (420 nm < λ < 780 nm) was used as the light source. At given intervals (every 30 minutes), the concentration of toluene in the reactor was measured with a GC-FID (FULI 9790, China). The schematic of the photocatalytic reactor was provided in the supporting information (see Fig. S1).

## Results and Discussion

### Structure and morphology

**Figure 1:**
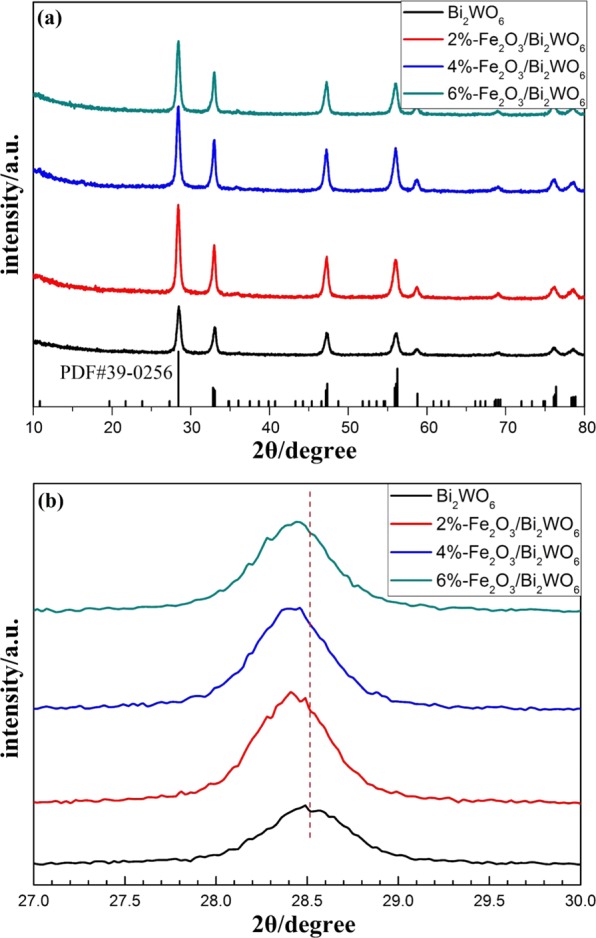
(**a**) XRD patterns of pure Bi_2_WO_6_ and Fe_2_O_3_/Bi_2_WO_6_ composites. (**b**) Enlargement of XRD peaks in lattice (131) plane.

The crystal structure and phase composition of these samples were detected by XRD analysis (Fig. 1a). For pure Bi_2_WO_6_, distinct diffraction peaks at 28.5^°^, 32.8^°^, 47.1^°^, 55.7^°^, 58.5^°^ and 75.8^°^ were perfectly corresponding to (131), (200), (202), (133), (262) and (391) crystallographic planes (PDF#39-0256)^27^, respectively. In addition, all the diffraction peaks of pure α-Fe_2_O_3_ (see Fig. S2) were in good agreement with those for hematite (PDF#33-0664)^28^. The intensity of the diffraction peaks of α-Fe_2_O_3_/Bi_2_WO_6_ composites was stronger than that of pure Bi_2_WO_6_ (see Fig. 1a), which could be ascribed to the growth of crystals during the hydrothermal process. It was noteworthy that no peaks of α-Fe_2_O_3_ were observed for α-Fe_2_O_3_/Bi_2_WO_6_ composite photocatalysts, which may be ascribed to the high dispersion and low content of α-Fe_2_O_3_^29^. Moreover, a shift to lower 2θ value of the band related to (131) lattice plane for α-Fe_2_O_3_/Bi_2_WO_6_ could be observed (Fig. 1b). Based on Bragg’s law, this fact verified the slight expansion of the interplanar spacing related to Bi_2_WO_6_^30^. According to the reference^31^, the increase in d spacing of Bi_2_WO_6_ could be attributed to the partial substitution of Bi sites by Fe ions. From this viewpoint, it was confirmed that there existed strong interaction between α-Fe_2_O_3_ and Bi_2_WO_6_. Similar results were also reported by the reference^25^.

**Figure 2:**
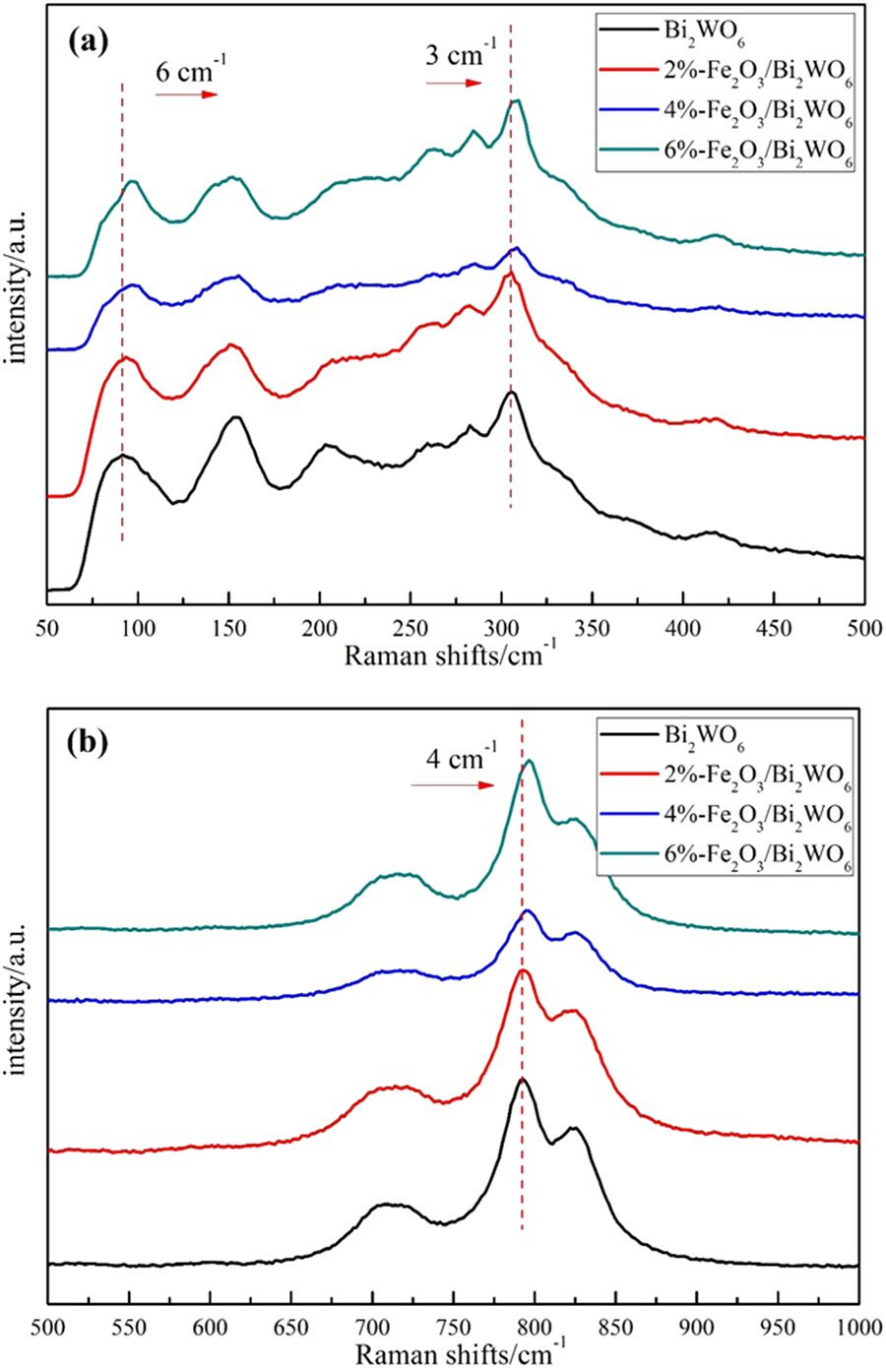
Raman spectra for pure Bi_2_WO_6_ and Fe_2_O_3_/Bi_2_WO_6_ composites in the range of (**a**) 50–500 cm^−1^ and (**b**) 500–1000 cm^−1^.

Raman spectra were also collected to further analyze the phase structure of photocatalysts. In Fig. 2, the peaks in the 60–160 cm^−1^, 200–400 cm^−1^ and 600–1000 cm^−1^ regions could be assigned to translational motions of Bi^3+^ and W^6+^, WO_6_ bending modes and Bi-O stretching and bending modes, and W–O bands stretching modes^32^, respectively. In detail, the bands at about 95 cm^−1^ and 305 cm^−1^ were associated with translational modes involving simultaneous motions of Bi^3+^ and WO_6_^6–33^. The peak at about 710 cm^−1^ could be ascribed to an antisymmetric bridging mode of the tungstate chain^33,34^. Two bands at about 790 cm^−1^ and 820 cm^−1^ matched well with antisymmetric and symmetric A_g_ modes of terminal O–W–O groups^33,35^. No Raman vibrational peaks of α-Fe_2_O_3_ were detected, which may be due to a lower α-Fe_2_O_3_ loading^29^. As illustrated in Fig. 2a,b, three bands at 95 cm^−1^, 305 cm^−1^ and 790 cm^−1^ of the α-Fe_2_O_3_/Bi_2_WO_6_ composites migrated to higher wave numbers, indicating the *in situ* growth of α-Fe_2_O_3_ on Bi_2_WO_6_ crystal had an influence on the phase structure of Bi_2_WO_6_. This finding fitted well with the results obtained from XRD characterization above.

**Figure 3:**
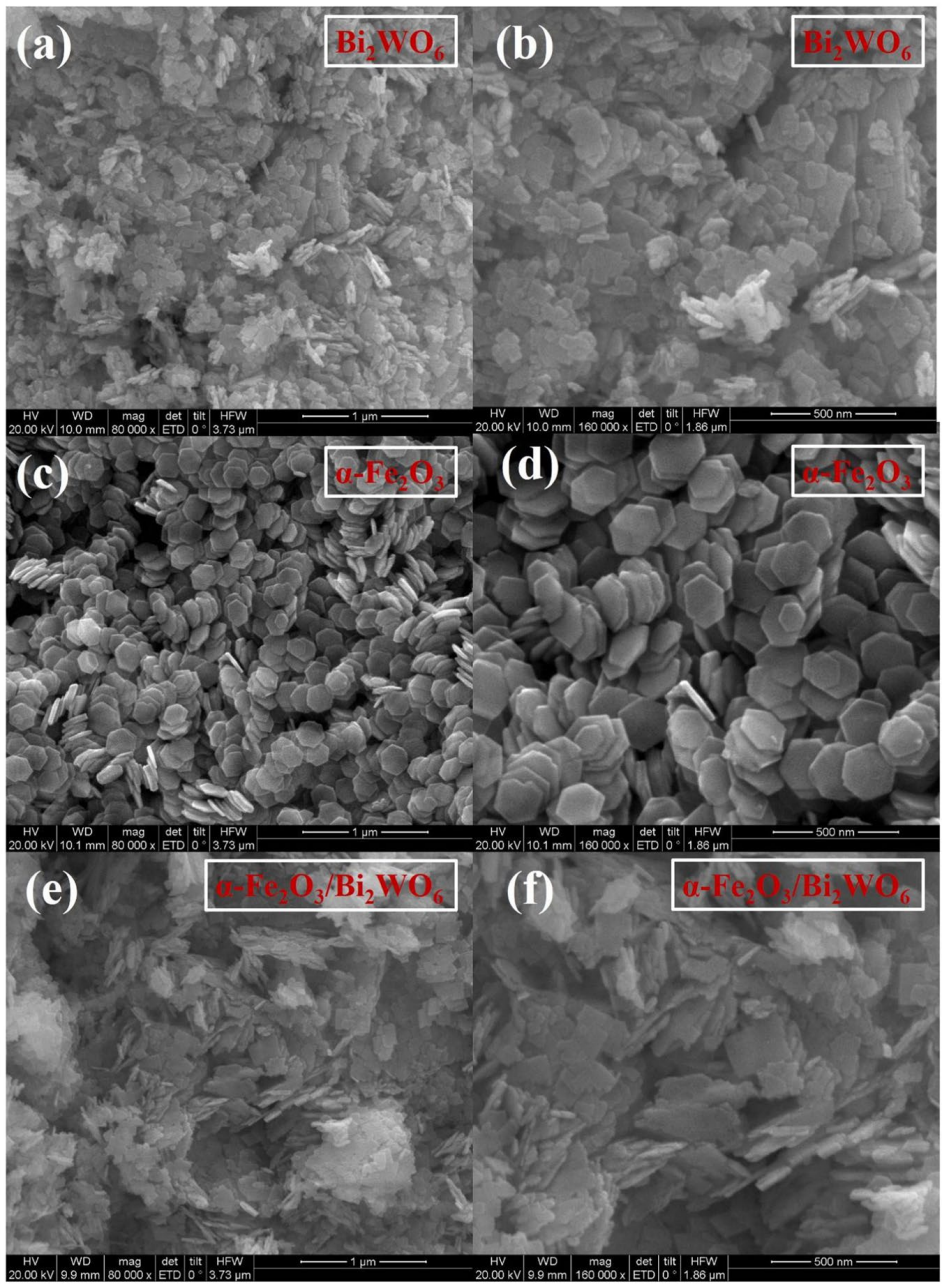
SEM images of (**a**,**b**) pure Bi_2_WO_6_, (**c**,**d**) pure α-Fe_2_O_3_ and (**e,f**) 4%-Fe_2_O_3_/Bi_2_WO_6_ composite.

**Figure 4:**
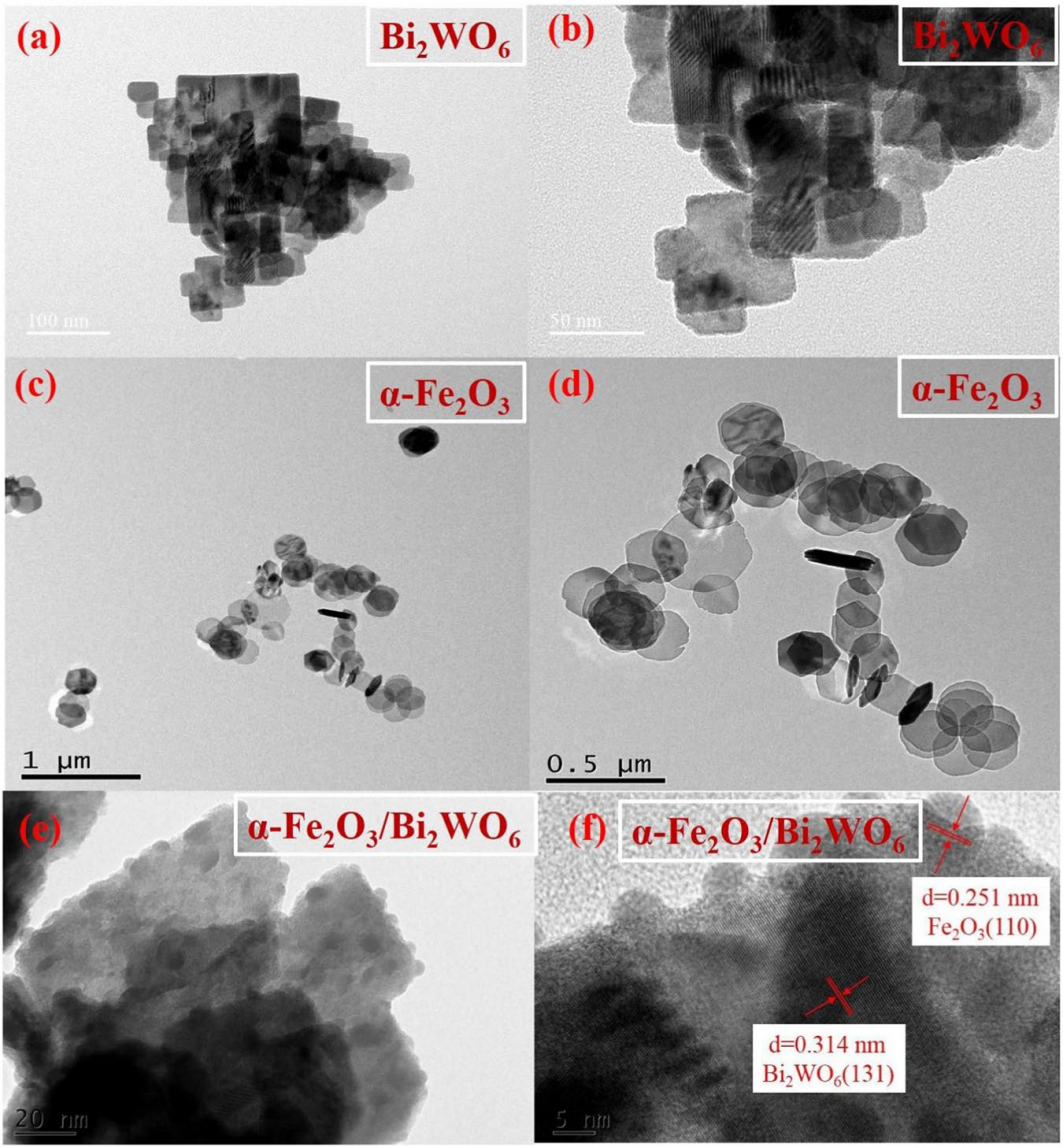
TEM images of pure (**a**,**b**) Bi_2_WO_6_, (**c**,**d**) pure α-Fe_2_O_3_, (**e**) TEM and (**f**) HR-TEM images of 4%-Fe_2_O_3_/Bi_2_WO_6_ composite.

The microstructure and morphology of the obtained samples were visualized by SEM, TEM and HR-TEM measurements. Figure 3 showed the SEM images of pure Bi_2_WO_6_, pure α-Fe_2_O_3_ and 4%-Fe_2_O_3_/Bi_2_WO_6_ composite, respectively. It could be clearly seen that the geometric shape of pure Bi_2_WO_6_ was a laminated structure (Fig. 3a,b). Pure α-Fe_2_O_3_ had a uniform and hexagonal nanoplate structure (Fig. 3c,d). No significant change could be observed in the SEM images of 4%-Fe_2_O_3_/Bi_2_WO_6_ composite (Fig. 3e,f) compared with that of pure Bi_2_WO_6_, indicating the prepared process hardly damaged the origin layered structure of pure Bi_2_WO_6_. Additionally, no hexagonal nanoplate structure of α-Fe_2_O_3_ could be found as well, which may be ascribed to the low content of α-Fe_2_O_3_^29^. The TEM images of pure Bi_2_WO_6_ (Fig. 4a,b) and pure α-Fe_2_O_3_ (Fig. 4c,d) showed layered structure as well. Figure 4e displayed that α-Fe_2_O_3_ nanosheets randomly grew on the laminated structure of Bi_2_WO_6_. A typical HR-TEM image of α-Fe_2_O_3_/Bi_2_WO_6_ composite was given in Fig. 4f. The lattice with interplanar distances of 0.314 nm and 0.251 nm attached to (131) lattice plane of Bi_2_WO_6_^30^ and (110) lattice plane of Fe_2_O_3_^26^, respectively. In view of long-time ultrasonication pretreatment in the TEM process, it could be concluded that there existed strong interlayer interaction between α-Fe_2_O_3_ and Bi_2_WO_6_ nanoplates^36^.

### Surface composition analysis

**Figure 5:**
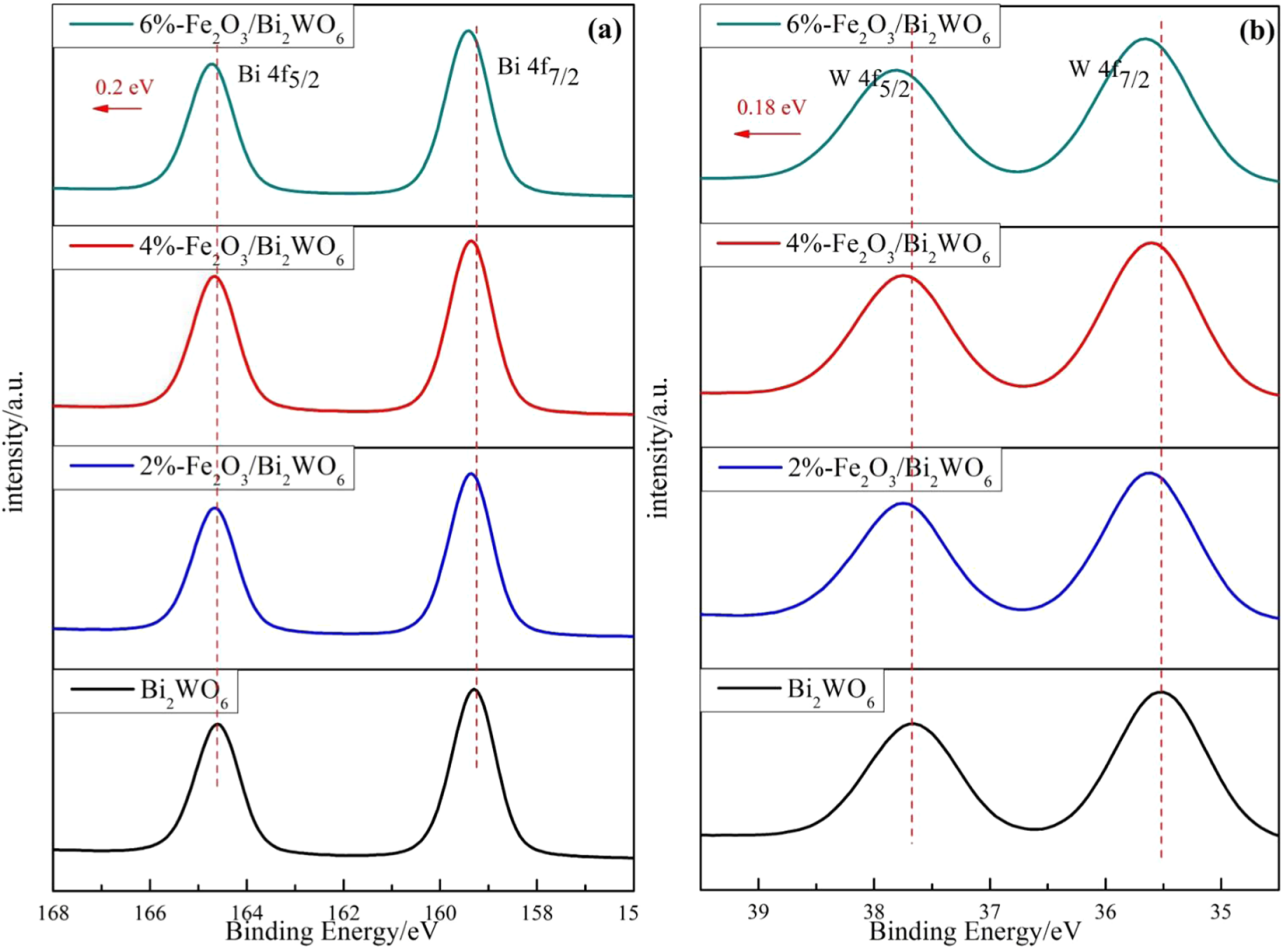
High-resolution XPS spectra of (**a**) Bi 4f (**b**) W 4f for different photocatalysts.

X-ray photoelectron spectroscopy (XPS) measurement was used to identify the oxidation state and surface composition of α-Fe_2_O_3_/Bi_2_WO_6_ composites. For pure Bi_2_WO_6_, two peaks with binding energy of 164.60 eV and 159.25 eV were the split signals of Bi 4f (Fig. 5a), which could be assigned to the Bi^3+^ species in the sample^37^. Two characteristic peaks in the W 4f spectrum (Fig. 5b) at 37.70 eV and 35.50 eV were ascribed to W 4f_5/2_ and W 4f_7/2_^37^, respectively. As displayed in Fig. S3, the peaks at 724.58 eV and 710.68 eV belonged to Fe 2p_1/2_ and Fe2p_3/2_^38^, suggesting the presence of Fe^3+^. The peaks of Bi^3+^ 4f and W^6+^ 4f both showed a slightly positive shift (see Fig. 5), which indicated the surface electron density for Bi and W had changed^39,40^. This result indicated that the heterostructure interface between Fe_2_O_3_ and Bi_2_WO_6_ could have been formed^15,36^. It agreed well with the abovementioned XRD, Raman and HR-TEM results.

### Optical properties

**Figure 6:**
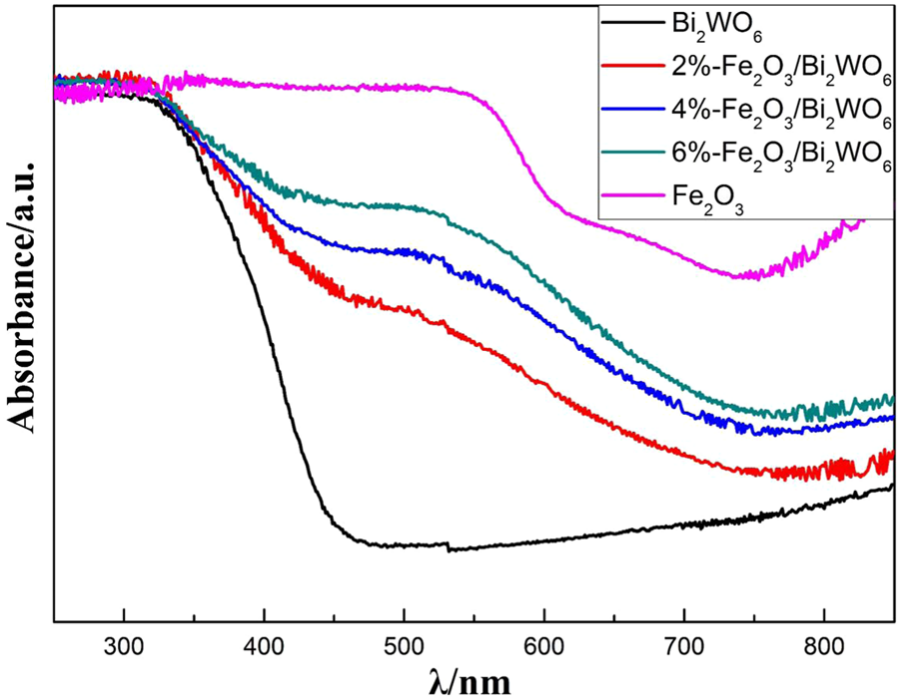
UV−vis absorption spectra of pure Bi_2_WO_6_, pure α-Fe_2_O_3_ and α-Fe_2_O_3_/Bi_2_WO_6_ composites.

Expanding the visible light absorption range played a crucial role for the modification of photocatalysts towards improving the visible-light photocatalytic activity. Optical diffuse reaction spectra (DRS) was used to estimate the visible light adsorption property of as-obtained photocatalysts. As shown in Fig. 6, pure α-Fe_2_O_3_ displayed an excellent light absorption ability among almost the whole visible light range due to its narrow band gap^41^. The spectrum of α-Fe_2_O_3_/Bi_2_WO_6_ composites showed obvious red-shift compared with pure Bi_2_WO_6_, implying the improvement of the utilization of visible light^42^. Furthermore, the band gap of pure Bi_2_WO_6_ and pure Fe_2_O_3_ was calculated based on Kubelka–Munk theory to be about 2.65 eV and 1.95 eV, respectively (see Fig. S4). These results agreed well with the references^43,44^.

### Photoelectrochemical performances

**Figure 7:**
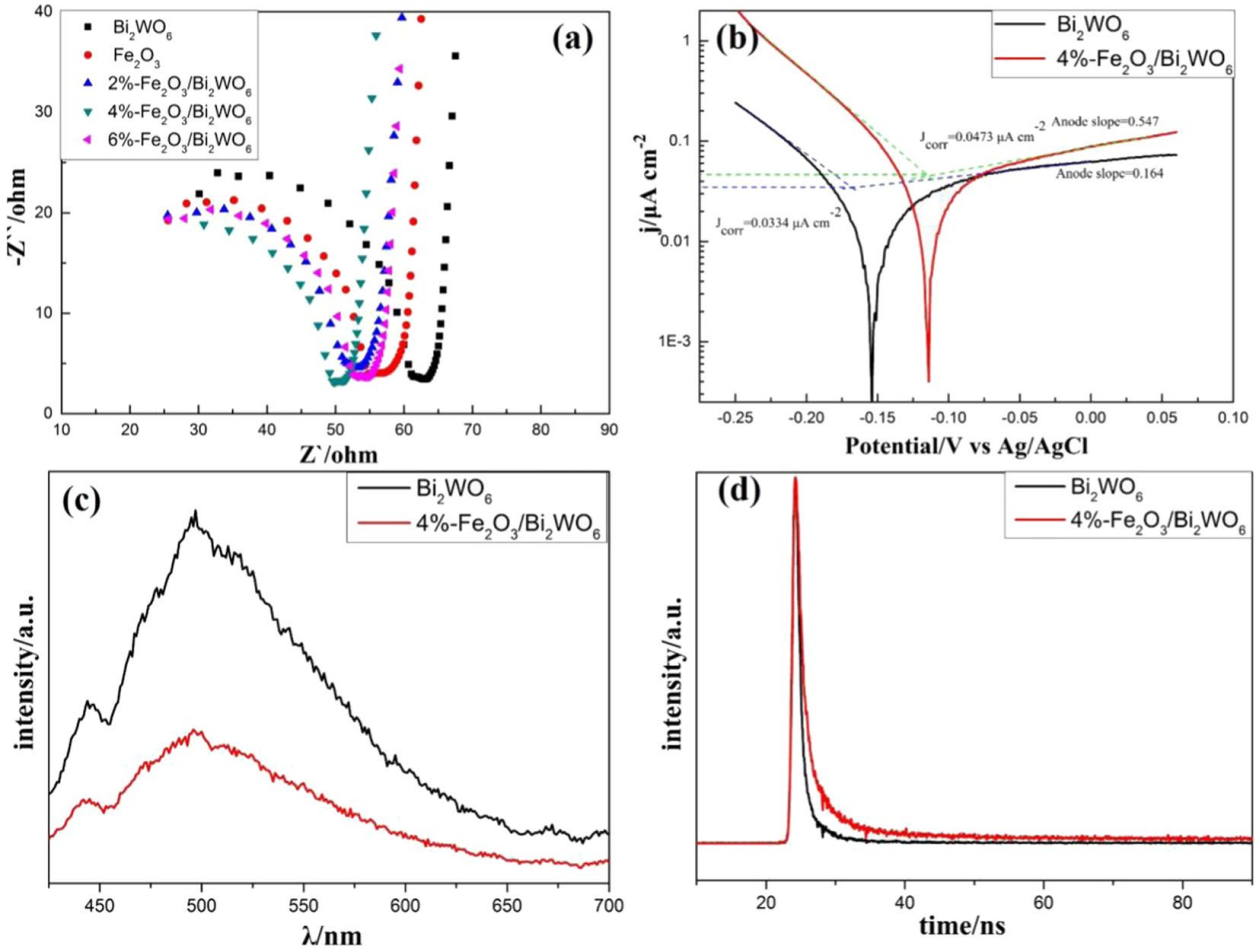
(**a**) Electrochemical impedance spectroscopy spectra of different catalysts, (**b**) Tafel polarization curves of the various electrodes under visible light irradiation, (**c**) Photoluminescence (PL) spectra and (**d**) time-resolved photoluminescence (TRPL) spectra of different samples.

Electrochemical impedance spectroscopy (EIS) was applied to shed light on the interface charge separation and transfer efficiency of different photocatalysts under visible light irradiation. The arc radius of Fe_2_O_3_/Bi_2_WO_6_ composites was smaller than that of pure Bi_2_WO_6_ and Fe_2_O_3_(see Fig. 7a), revealing that the photoexcited eletrons could easily transfer across the interface and efficiently migrate to the surface due to the Fe_2_O_3_/Bi_2_WO_6_ layered heteojunction^39,45^. Especially, the 4%-Fe_2_O_3_/Bi_2_WO_6_ composite had the smallest arc radius, indicating it possessed the least electrons transfer resistance. Tafel analysis was performed to get a vivid view of the current density values of the photocatalysts investigated under visible light irradiation^46^. Generally, larger values of corrosion current density and anodic Tafel slope meant more photogenerated pairs and faster electron transfer rate^12^, which benefitting the photocatalytic process. As displayed in Fig. 7b, 4%-Fe_2_O_3_/Bi_2_WO_6_ composite exhibited larger J_corr_ value and anodic slope as compared with pure Bi_2_WO_6_. Consequently, the results further demonstrated that the heterojunction between Fe_2_O_3_ and Bi_2_WO_6_ could improve charge transfer rate and efficiently separate photoexcited electrons and holes. Moreover, as shown in Fig. S5, the photocurrent of Fe_2_O_3_/Bi_2_WO_6_ composite was much higher than that of pure Bi_2_WO_6_, suggesting the separation ratio of photoexcited charges enhanced. Photoluminescence (PL) spectra was further used to demonstrate the recombination ratio of photoinduced pairs. As displayed in Fig. 7c, the intensity of 4%-Fe_2_O_3_/Bi_2_WO_6_ was much lower than that of pure Bi_2_WO_6_, indicating that the recombination of photogenerated charge carriers was effectively inhibited^15,47^. Time-resolved photoluminescence (TRPL) spectra (Fig. 7d) was also used to assess recombination kinetics of photoinduced electron-hole pairs. The TRPL decay spectrum waves were fitted by exponential decay kinetics function displayed as Eq. (1) ^48^:

1 $$I(t)={{\rm{A}}}_{1}\,\exp (\,-\,{\rm{t}}/{{\rm{\tau }}}_{1})+{{\rm{A}}}_{2}\,\exp (\,-\,{\rm{t}}/{{\rm{\tau }}}_{2})$$

The average emission time was calculated based on Eq. (2) ^48^:

2 $${{\rm{\tau }}}_{{\rm{av}}}=\sum {{\rm{A}}}_{{\rm{i}}}{{{\rm{\tau }}}_{{\rm{i}}}}^{2}/\sum {{\rm{A}}}_{{\rm{i}}}{{\rm{\tau }}}_{{\rm{i}}}$$

where τ_1_ and τ_2_ were lifetimes, A_1_ and A_2_ were the corresponding weighting factors. Based on the fitted results listed in Table 1, the average lifetime of 4%-Fe_2_O_3_/Bi_2_WO_6_ composite was longer than that of pure Bi_2_WO_6_, enhancing the likelihood of photoinduce pairs to participate in the reaction of gas-phase toluene photodegradation^39^, thereby implying higher photocatalytic activity for Fe_2_O_3_/Bi_2_WO_6_ composites.

**Table 1:** Kinetic parameters of the emission Decay of different samples.

Sample	τ_1_/ns	A_1_/%	τ_2_/ns	A_2_/%	χ^2^	τ_av_/ns
Bi_2_WO_6_	0.7402	63.41	3.5458	36.59	1.407	2.8005
4%-Fe_2_O_3_/Bi_2_WO_6_	1.2237	60.52	5.8734	39.48	1.186	4.7478

### Photocatalytic performances

**Figure 8:**
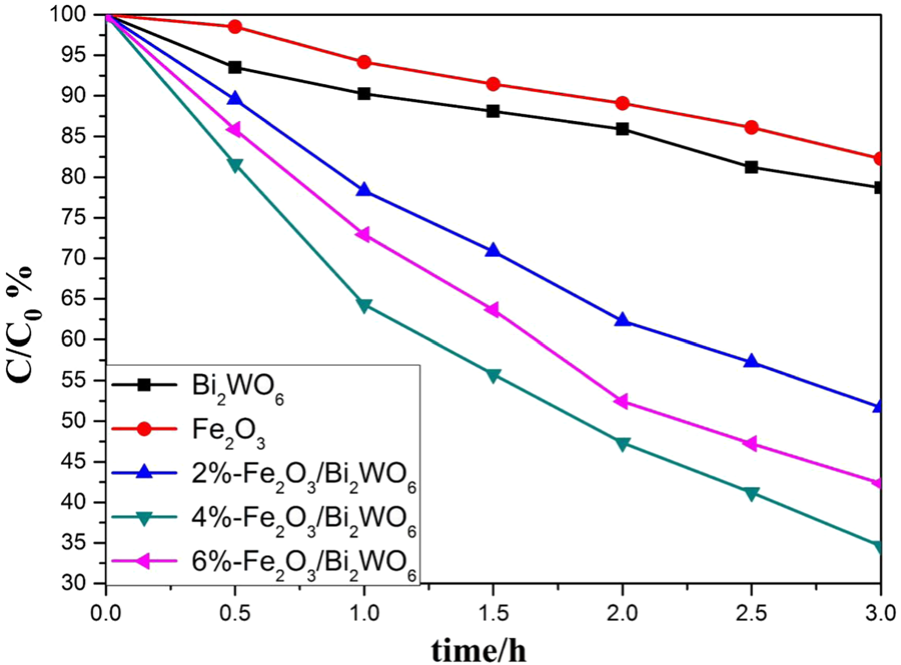
Photocatalytic degradation of gaseous toluene in the presence of different samples under visible light irradiation.

To evaluate the photocatalytic efficiency of as-prepared photocatalysts, gaseous toluene was chosen as a probe indoor air contaminant. The toluene degradation efficiency for the samples investigated was displayed in Fig. 8 and the relative apparent rate constant (*k*) based on pseudo-first-order kinetic model was obtained (see Fig. S6). Pure Bi_2_WO_6_ and α-Fe_2_O_3_ showed rather poor photocatalytic activity under three-hour visible light irradiation. In stark contrast, the gaseous toluene removal efficiency was remarkedly enhanced over Fe_2_O_3_/Bi_2_WO_6_ composites owing to their well photoelectrochemical property as discussed above. Among all the samples, 4%-Fe_2_O_3_/Bi_2_WO_6_ catalyst showed the highest photocatalytic activity, whose *k* value (0.3469 h^−1^) was much higher than those of pure Bi_2_WO_6_ (0.0749 h^−1^) and pure α-Fe_2_O_3_ (0.0649 h^−1^). Besides, the surface area didn’t play an important role in enhancing the photocatalytic activity, as the surface area of 4%-Fe_2_O_3_/Bi_2_WO_6_ sample was modest (see Table S1). Based on the above analysis, it could be concluded that the layered heterojunctions between Fe_2_O_3_ and Bi_2_WO_6_ palyed a key role in promoting the photocatalytic process.

### Possible photocatalytic mechanism

**Figure 9:**
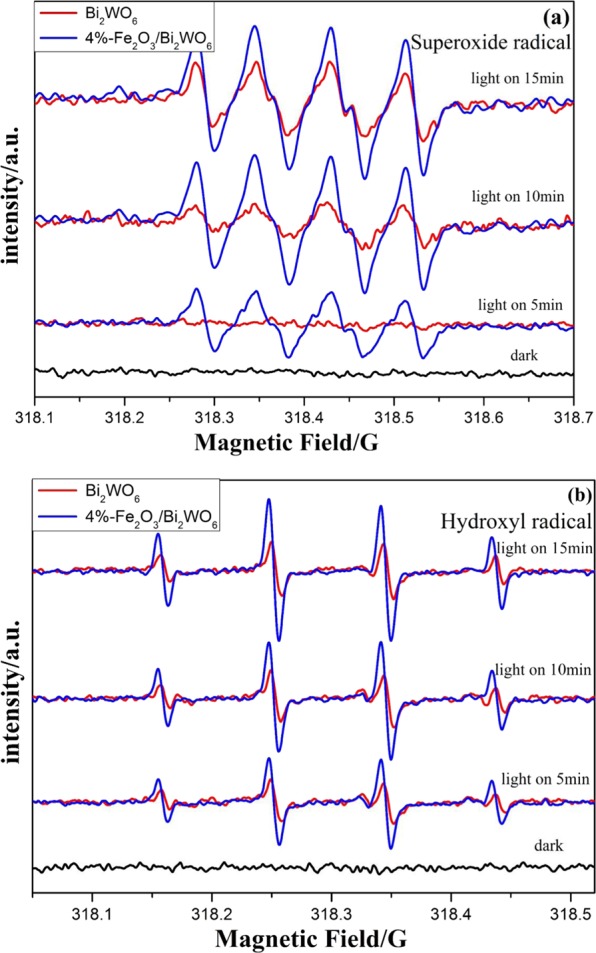
DMPO spin-trapping ESR spectra in Bi_2_WO_6_ and 4%-Fe_2_O_3_/Bi_2_WO_6_ composite in drak or under visible light irradiation: (**a**) in aqueous dispersion for DMPO-**·**O_2_^−^ and (**b**) in methanol dispersion for DMPO-**·**OH.

**Figure 10:**
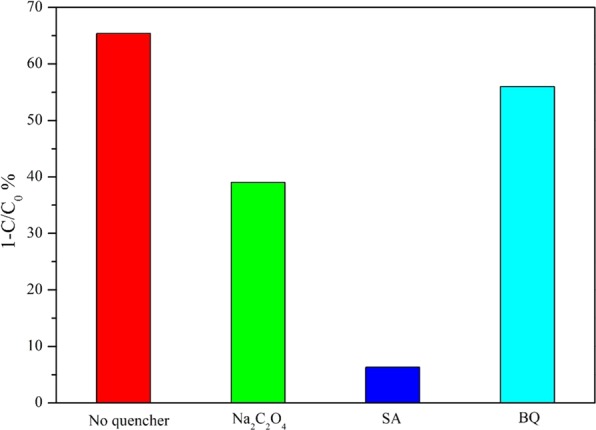
Photocatalytic activities of 4%-Fe_2_O_3_/Bi_2_WO_6_ composite for the photooxidation of toluene in the presence of different quenchers.

DMPO spin-trapping ESR technique was employed to verified the active radicals produced in the photocatalytic system. As presented in Fig. 9, no peaks could be observed in the darkness. Clearly, characteristic peaks for **·**OH and **·**O_2_^−^ emerged once the as-obtained samples were irradiated by visible light. Both intensity for **·**OH and **·**O_2_^−^ became stronger with increasing irradiation time. Notably, the 4%-Fe_2_O_3_/Bi_2_WO_6_ exhibited stronger intensity for **·**OH and **·**O_2_^−^ than that of pure Bi_2_WO_6_ under the same condition, implying the oxidation ability of the composite photocatalyst had been effectively promoted^49^. As reported in the references^50–52^, the involvement of active radical species (such as **·**O_2_^−^, **·**OH and h^+^) was very important in the photocatalytic process. Thus, free radicals trapping experiment was performed to further figure out the role of the radical species during the photocatalytic reaction. In a typical experiment, Na_2_C_2_O_4_^53^, salicylic acid (SA)^54^ and benzoquinone (BQ)^55^ were applied as scavengers of h^+^, **·**OH and **·**O_2_^−^, respectively. As illustrated in Fig. 10, the addition of BQ had little effect on the photocatalytic activity. However, the toluene removal efficiency obviously decreased in the presence of Na_2_C_2_O_4_ and SA. In particular, the degradation efficiency of toluene was whittled down into about 5% when adding appropriate amount of SA. Therefore, it could be inferred that h^+^ and **·**OH played dominant roles in the toluene photodegradation process.

**Figure 11:**
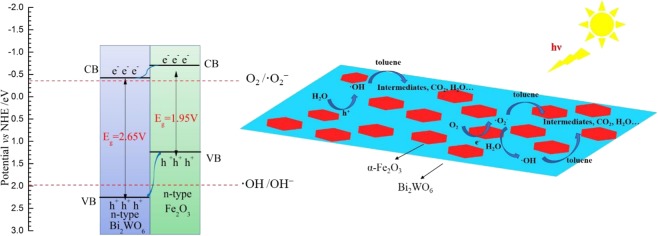
Diagram of the energy band levels of Fe_2_O_3_/Bi_2_WO_6_ composite and proposed possible photodegradation of toluene process.

Based on the Mott-Schottky analysis (see Fig. S7), the value of Fermi energy level was −0.30 eV and −0.60 eV (*vs* NHE) for Bi_2_WO_6_ and α-Fe_2_O_3_^56^, respectively. For n-type semiconductor, the conduction band (CB) edge was more negative (about 0–0.2 eV) than Fermi level^57^. Herein, the difference value was set to 0.1 eV. Therefore, as illustrated in Fig. 11, the conduction band (CB) bottom and valence band (VB) top values of pure Bi_2_WO_6_ and pure α-Fe_2_O_3_ could be obtained. The *in situ* growth strategy could guarantee the intimate contact between Bi_2_WO_6_ and α-Fe_2_O_3_ with stronger interfacial interaction. Once the heterojunction was irradiated by visible light, both Bi_2_WO_6_ and α-Fe_2_O_3_ could generate electron-hole pairs. Due to the type-II heterojunction and intimate contact between Bi_2_WO_6_ and α-Fe_2_O_3_, the photoexcited electrons on the CB of α-Fe_2_O_3_ had the tendency to transfer to that of Bi_2_WO_6_, whereas photoinduced holes spontaneously moved to the VB of α-Fe_2_O_3_ (see Fig. 11). Therefore, the photogenerated electrons and holes could be separated efficiently, which greatly enhanced the photocatalytic activity. The CB bottom level of Bi_2_WO_6_ was more negative than that of O_2_/**·**O_2_^−^ (−0.33 eV)^58^, thus the photoexcited electrons could reduce oxygen molecules absorbed on the surface to **·**O_2_^−^ species. What’s more, part of O_2_ could be reduced to H_2_O_2_ by photo-generated electrons based on the fact that the redox potential of O_2_/H_2_O_2_ was 0.695 eV^59^, then the formed H_2_O_2_ could produce **·**OH by capture the photo-generated electrons^60,61^. Although the VB top value of α-Fe_2_O_3_ was less positive than the potential of **·**OH/OH^−^ (1.99 eV)^58^, characteristic signals of **·**OH (1:2:2:1 quartet pattern) could still be obviously observed in the ESR spectrum (see Fig. 9). This may be ascribed to the fact that part of the photogenerated holes remaining in the VB top of Bi_2_WO_6_ could also oxidize absorbed H_2_O into **·**OH. Similar phenomenon was also founded by Li. *et al*.^39^. Hence, these radical species had strong oxidation ability to destroy toluene molecule.

## Conclusions

In summary, α-Fe_2_O_3_/Bi_2_WO_6_ layered heterojunctions were successfully synthesized via a simple *in situ* growth method in this work. XRD, Raman, HR-TEM and XPS results demonstrated there existed strong interaction between Bi_2_WO_6_ and α-Fe_2_O_3_. The α-Fe_2_O_3_/Bi_2_WO_6_ layered heterojunctions could effectively broaden visible light absorption range and improve photoexcited charges separation efficiency according to the characterization results of UV-vis, EIS, Tafel curve, PL and TRPL. Especially, 4%-Fe_2_O_3_/Bi_2_WO_6_ sample with strong interlayer interaction exhibited the highest photocatalytic activity. Given the results of ESR and trapping experiments, h^+^ and **·**OH species played a crucial role during the photocatalytic process of toluene removal. Such layered heterojunction photocatalyst had potential applications for indoor air purification.

## Supplementary information

surpporting information
