# Supplementary material for: Synthesis of α-Fe2O3/Bi2WO6 layered heterojunctions by in situ growth strategy with enhanced visible-light photocatalytic activity
Source: Sci Rep. 2019 May 17;9:7551. doi: 10.1038/s41598-019-43917-w (PMC6525269; doi:10.1038/s41598-019-43917-w)
Supplement: Supplementary file 1 — surpporting information [file 41598_2019_43917_MOESM1_ESM.docx]

**Synthesis of α-Fe_2_O_3_/Bi_2_WO_6_ layered heterojunctions by *in situ* growth strategy with enhanced visible-light photocatalytic activity**

Taiping Xie^1^, Yue Liu^1^*, Haiqiang Wang ^1^ and Zhongbiao Wu^1,2^

^1^Department of Environmental Engineering, Zhejiang University, 866 Yuhangtang Road, Hangzhou, 310058, P. R. China.

^2^Zhejiang Provincial Engineering Research Center of Industrial Boiler & Furnace Flue Gas Pollution Control, 866 Yuhangtang Road, Hangzhou, 310058, P. R. China.

*Corresponding author: Tel: +86 571 87953088; Fax: +86 87953088.

E-mail address: [yueliu@zju.edu.cn](mailto:yueliu@zju.edu.cn)


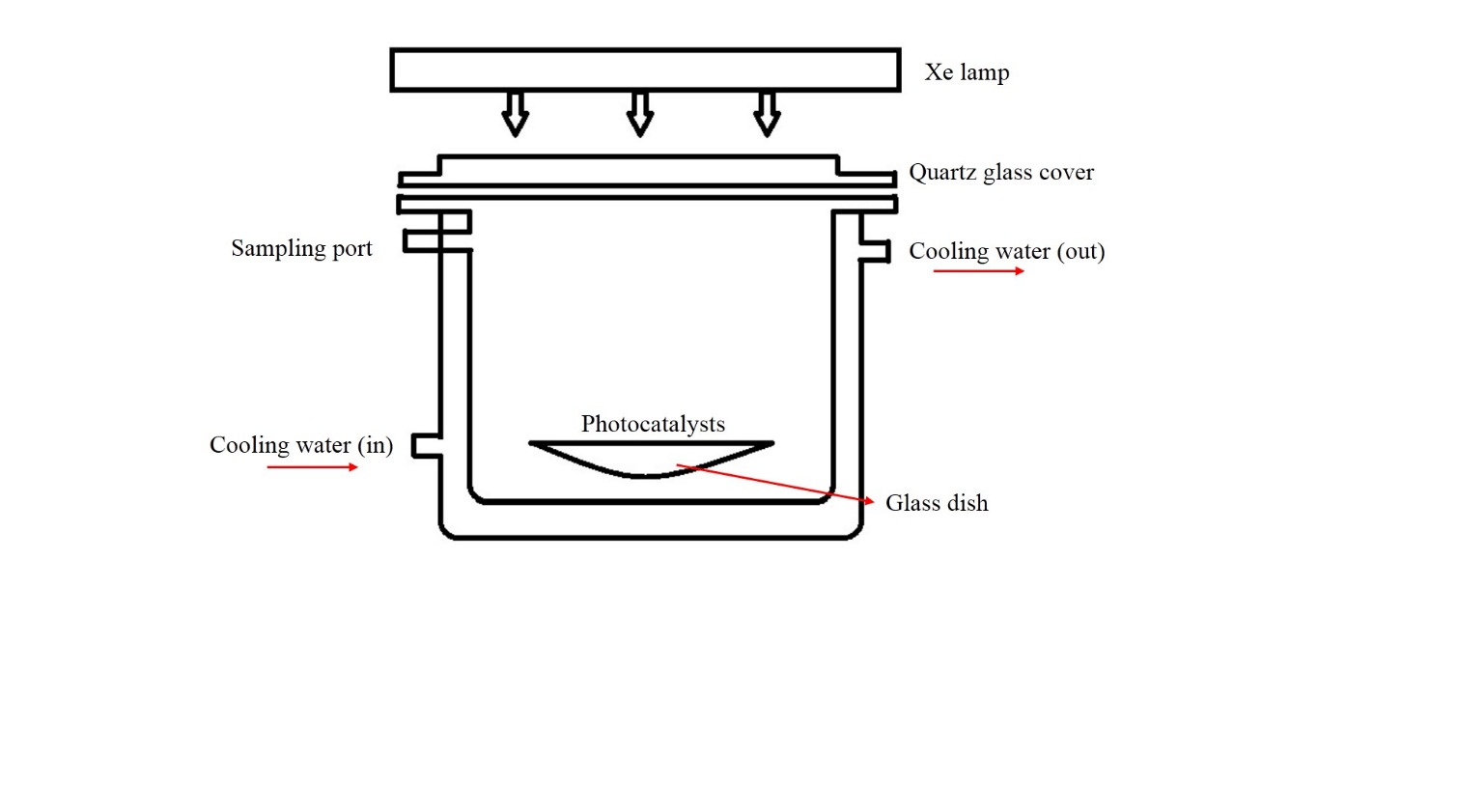


Fig. S1 The Schematic diagram of photocatalytic reactor for toluene removal


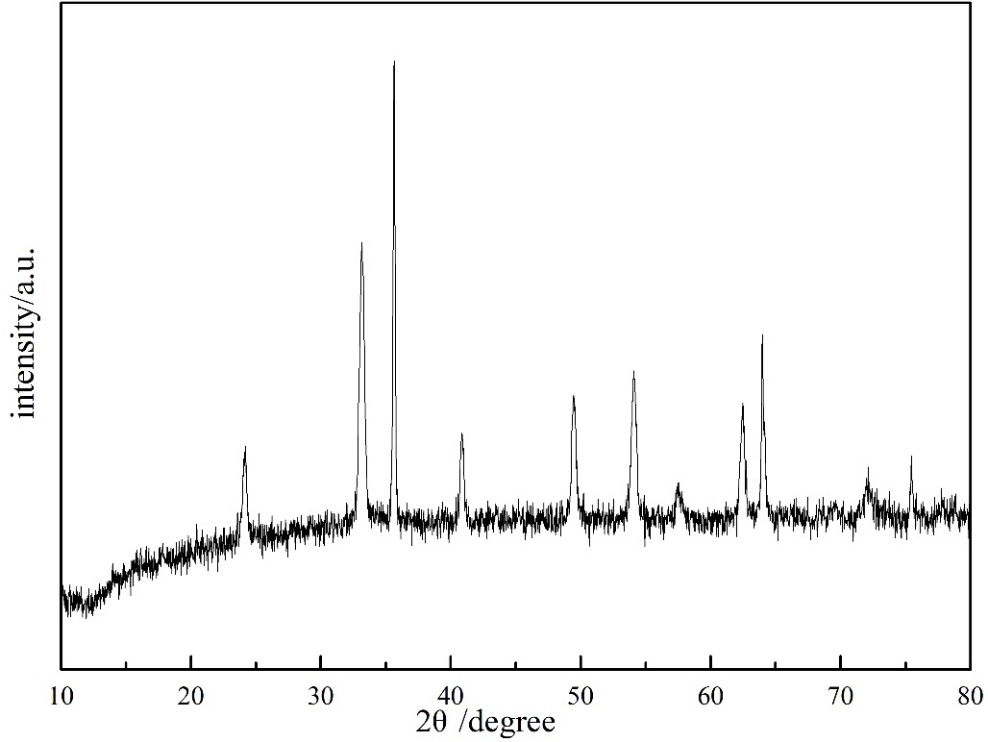


Fig. S2 XRD patterns of pure α-Fe_2_O_3_

_
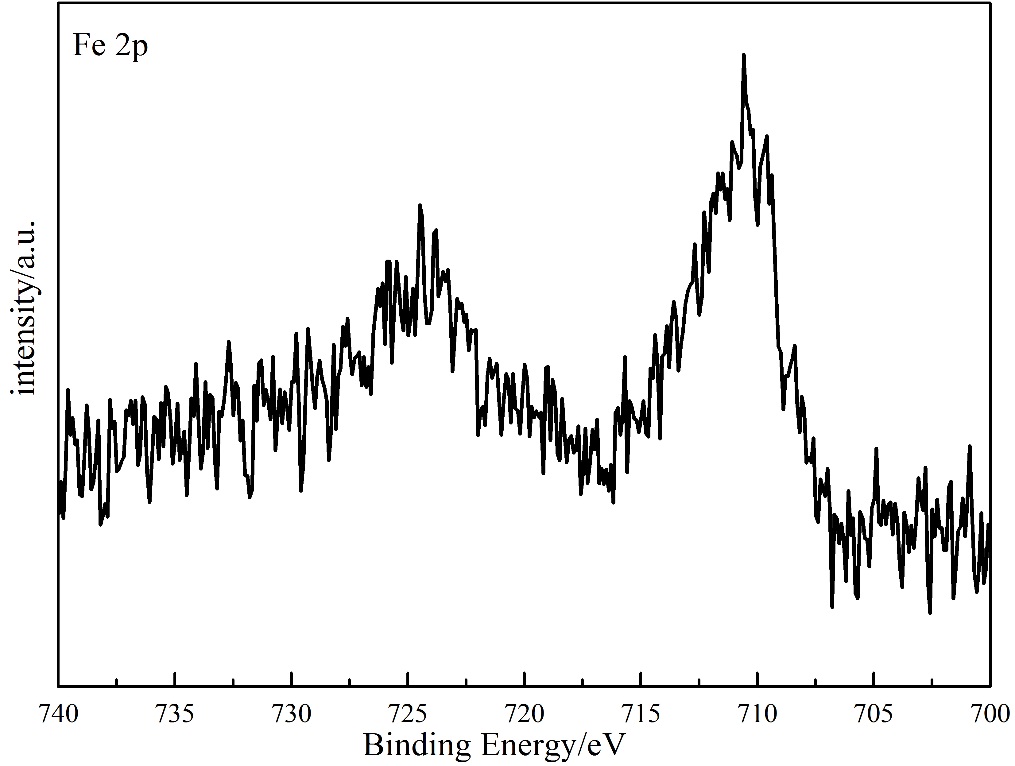
_

Fig. S3 XPS spectra of Fe 2p for 4%-Fe_2_O_3_/Bi_2_WO_6_ composite


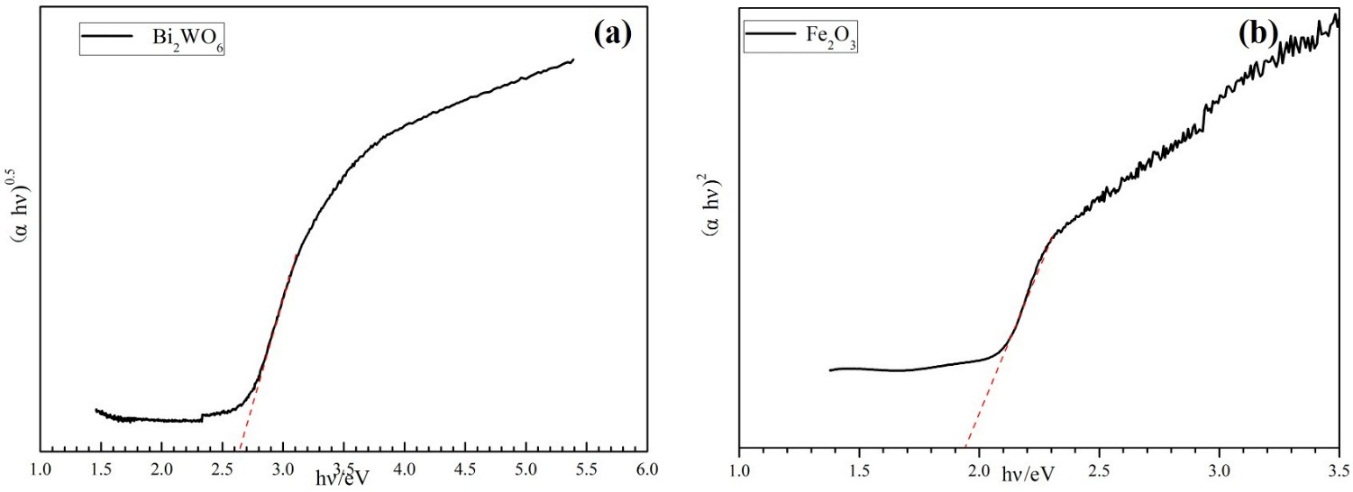


Fig. S4 (a) The plot of (αhν)^0.5^ versus photon energy (hν) of pure Bi_2_WO_6_, (b) the plot of (αhν)^2^ versus photon energy (hν) of pure α-Fe_2_O_3_


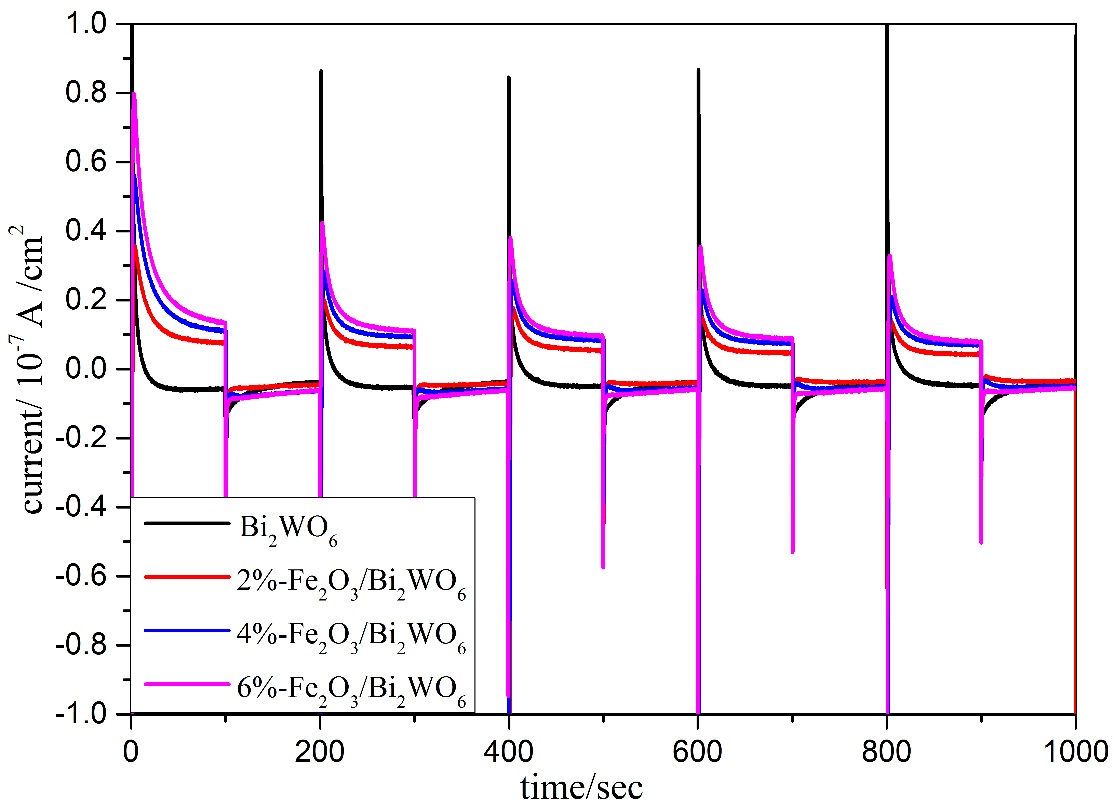


Fig. S5 Transient photocurrents of different samples


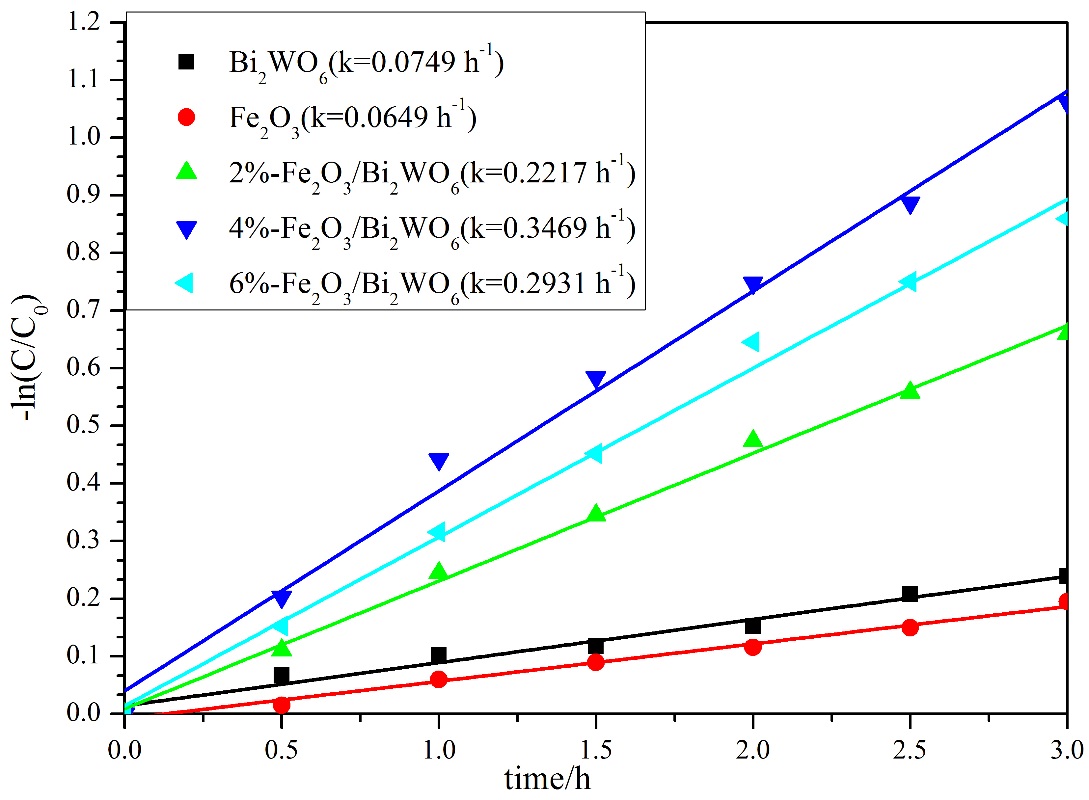


Fig. S6 First-order kinetics plot for the photodegradation of toluene on the as-obtained catalysts


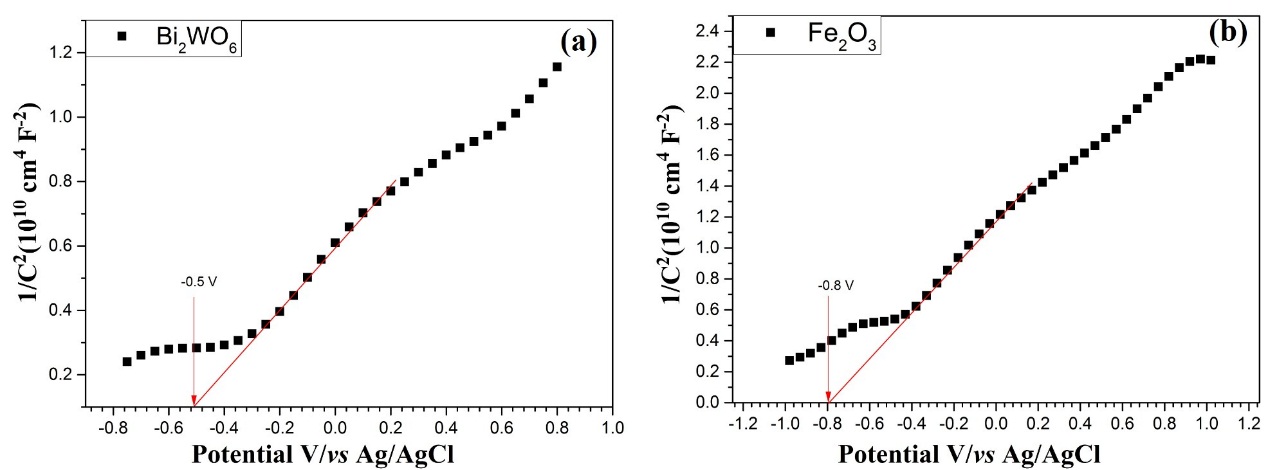


Fig. S7 Mott–Schottky plots collected for (a) pure Bi_2_WO_6_ and (b) α-Fe_2_O_3_ at a frequency of 1000 Hz under visible light irradiation

**Table S1** BET surface area and pore structure for the obtained catalysts

| Sample | Surface area (m^2^/g) | Pore volume (cm^3^/g) | Pore size (nm) |
| --- | --- | --- | --- |
| Bi_2_WO_6_ | 24.4 | 0.183 | 9.15 |
| 2%-α-Fe_2_O_3_/Bi_2_WO_6_ | 31.2 | 0.181 | 8.09 |
| 4%-α-Fe_2_O_3_/Bi_2_WO_6_ | 36.8 | 0.233 | 7.11 |
| 6%-α-Fe_2_O_3_/Bi_2_WO_6_ | 41.7 | 0.209 | 7.98 |
